# Special Issue “Selected Papers from the 16th Weurman Flavour Research Symposium”

**DOI:** 10.3390/molecules27113594

**Published:** 2022-06-02

**Authors:** Elisabeth Guichard, Jean-Luc Le Quéré

**Affiliations:** Centre for Taste, Smell and Feeding Behaviour (Centre des Sciences du Goût et de l’Alimentation, CSGA), INRAE, 21065 Dijon, France

## 1. Introduction

Since 1975, the Weurman Flavour Research Symposium has been held every three years in different European countries, and has been finally established as an international event that offers unique opportunities for distinguished scientists from academia and industry, from different disciplines, and from all over the world, to discuss trends and new paradigms in the field of flavour research.

Indeed, it is noteworthy that the research interest in the flavour field, nowadays including chemistry, biology, neuro-physiology and psychology, is still so active after almost 50 years. A new generation of enthusiastic scientists is already active to ensure the future of the research on flavour and perpetuate the tradition of this recognised international symposium, named in memory of Cornelius Weurman, who inspired the first edition held in the Netherlands.

Since the organisation of the first edition, obvious progress has been made in the understanding of the flavour composition of foods, the generation of flavour compounds and the biological mechanisms underlying flavour perception, involving the odour and taste receptors. Essentially dedicated to aroma compounds in its first age, flavour research now includes important contributions on taste, trigeminal and mouthfeel compounds, which allow researchers to display a more holistic picture of what is intended by the terms flavour and flavour perception. Moreover, new research areas, such as sensory interactions, perireceptor events, brain integration or relationships with food intake and well-being, have widened the domain of flavour research. If ‘traditional’ efforts on flavour analyses while developing new analytical tools and on flavour generation are still important, as reflected by the number of dedicated contributions during this 16th edition of the symposium, important contributions dealing with new paradigms were proposed to the scientific committee.

Therefore, the scientific programme of this 16th edition, held totally online on 4–6 May 2021, due to the COVID-19 crisis, and attended by 423 academic and industrial participants from 28 countries, covered the following main subject areas:

Multimodal flavour perception (from mixture of stimuli to brain integration);
Flavour perception: from molecules to receptors;
Flavour perception, food intake and well-being;
Flavour representation (naturality, authenticity);
Flavour generation;
Modelling flavour compounds activity;
New analytical tools for flavour analysis.

## 2. Highlights of the 16th Edition

The scientific programme (still accessible with the abstracts on the symposium website at https://symposium.inrae.fr/weurman2020/, accessed on 20 May 2022) comprised 194 presentations: 3 keynote lectures from experts in the field, 41 scientific talks given by distinguished scientists from academia and industry from around the world and a total of 150 posters, among which 18 were selected by the scientific committee as flash poster presentations. The presentations were organised in oral and poster sessions dedicated to:

Sensory interactions, introduced by a keynote lecture entitled “The question of Organic Unity in Flavour: The whole is not equal to the sum of the parts”, presented by Dr Thierry Thomas-Danguin (CSGA, INRAE, France).
Oral processing, release and perception.
Various subjects on flavour perception, including food intake and well-being aspects, introduced by a keynote lecture entitled “In defence of pleasure”, presented by Dr Dana Small (Yale University School of Medicine, USA), and the aspects covering the interplay between molecules and receptors, introduced by a keynote lecture entitled “Multiple sweet taste signalling pathways in taste cells”, presented by Dr Robert Margolskee (Monell Chemical Center, Philadelphia, PA, USA).
Flavour analyses and analytical tools.
Flavour generation.
Modelling (perception and flavour compounds activity).
The programme also comprised four workshops, with between 100 and 150 participants in each, on the following hot topics:
Chemistry and biochemistry of flavour compounds in the mouth: what we know.
Flavour perception: from molecules via bioreceptors to physiological functions.
In-vivo flavour release and perception by time-resolved sensory and instrumental methods.
Perceptual interactions in flavour perception.

The manuscripts gathered in the Special Issue are illustrative of the presentations given in the main sessions. Thus, two contributions focused on flavour perception.

Fischer et al. [1] studied the impact of ageing on pea protein volatile compounds and the correlation with pea protein odour. They found that the storage conditions had a profound impact on the volatile compounds, on the odour, and on the colour of the studied pea protein isolate (PPI). This work highlighted the importance of light exposition and the crucial role of the first three months of storage. Light exposition was detrimental to the colour and the odour of PPI, and a high increase in the total amount of volatile compounds was observed. A tentative correlation between instrumental data on volatile compounds and the perceived odour was proposed. The representation of volatile compounds sorted by their sensory descriptors allowed the researchers to predict odour changes with analytical data.

Wang et al. [2] studied the astringency sensitivity of a cohort of elderly and young people to tannic acid while taking into account the effect of ageing and saliva composition. They found that the astringency threshold was significantly higher in the elderly group than the young group. No correlation was observed between the salivary protein amount and threshold value. A negative correlation between salivary flow and threshold was observed in the young group only. They concluded that the observed difference can be linked to salivary properties that differ as a function of age.

Three contributions focused on flavour generation.

Bürger et al. [3] investigated the production of an anise- and woodruff-like aroma using monokaryotic strains of *Pleurotus sapidus* grown on citrus side streams. Their main conclusions highlighted the role of the character impact compound *p*-anisaldehyde in the anise- and woodruff-like note while the acyloin identified as (2S)-hydroxy-1-(4-methoxyphenyl)-1-propanone also contributed to the overall aroma. Supplementation of the culture medium with isotopically substituted L-tyrosine elucidated this amino acid as a precursor of *p*-anisaldehyde as well as of (2S)-hydroxy-1-(4-methoxyphenyl)-1-propanone, produced with an enantiomeric excess of 97% by *P. sapidus*.

Pisaniello et al. [4] studied the varietal influence of flavour precursors from grape marc on monoterpene and C_13_-norisoprenoid profiles in wine. With the use of an original analytical tool, membrane-assisted solvent extraction (MASE) GC-MS, they found that marc extracts produced from floral grape lots resulted in an increase in geraniol glucoside concentration when added to a base white wine after five to six months of storage. However, the volatile analysis after hydrolysis of the extracts did not provide useful information as to the profile of different monoterpenes, only the total magnitude. Thus, the addition of extracts to wine that were derived from varieties determined as ‘floral’ provided a greater increase in monoterpenes than ‘non-floral’ extracts.

Rigling et al. [5] investigated a robust fermentation process for natural chocolate-like flavour production with *Mycetinis scorodonius*. They found that submerged fermentation of green tea with the basidiomycete *M. scorodonius* resulted in a pleasant chocolate-like and malty aroma. Key aroma compounds were elucidated by GC-MS-O to be dihydroactinidiolide, isovaleraldehyde and coumarin, which were also confirmed by a recombination study. Variation in the fermentation parameters (medium volume, light protection, agitation rate, pH, temperature and aeration) were studied and up-scaling in volume resulted in longer growth cycles while aeration significantly boosted the concentrations.

Finally, three contributions focused on original analytical tools for flavour analysis.

Cong et al. [6] identified non-volatile compounds that impact flavour disliking of wholewheat bread made with aged flours using a dedicated untargeted HPLC-MS method. The chemical profiles of thirteen breads made from aged flours were obtained in a flavouromics approach and modelled by orthogonal partial least squares (OPLS) to predict flavour liking. They identified pinellic acid, 12,13-dihydroxy-9Z-octadecenoic acid and 1-(9Z,12Z-octadecadienoyl)-*sn-glycero*-3-phosphocholine as predictive chemical features and sensory analysis confirmed that the three compounds increased the bitterness intensity of the bread samples. The concentrations of all bitter compounds were found significantly higher in bread made from aged flour versus non-aged flour.

Rossignol et al. [7] developed a microwave sensor based on molecularly imprinted silica for the real-time detection of phenylacetaldehyde in wine. They concluded that the developed sensor based on molecularly imprinted sol–gel silica (MIS) coupled to a microwave sensor was able to detect phenylacetaldehyde (PAA), a chemical tracer of wine oxidation, at the µg/L level, which is below the off-flavour threshold of PAA in red wine.

Hayashi et al. [8] used a chewing simulator hyphenated to a PTR-MS instrument to analyse the retronasal aroma of beef pate. They were able to study the release of aroma compounds under controlled in-vitro mastication and salivation conditions. They could explain the effect of fat and soy protein contents on the temporal release of aroma compounds. Thus, the release of certain types of aromas was significantly faster with stronger chewing force, and higher with a high-fat content in the sample. Moreover, a larger release intensity was observed when soy proteins were partially substituted for beef proteins. This finding was judged important for the better reformulation of healthy foods with less fat and for the partial substitution of animal with plant proteins. In addition, the formulation of artificial saliva with varying levels of proteins or minerals made it possible to mimic the saliva composition of an elderly person. They found a change in the salting-out effect from the saliva composition as an important factor, which could explain the lowering of aroma release in an elderly person and contribute to a loss of perception.

The major part of the contributions may be found as short communications in the Proceedings e-book, available on the Zenodo platform at https://www.zenodo.org/communities/weurman2021/ (accessed on 20 May 2022). The 17th edition of the Weurman Flavour Research Symposium is scheduled for 24–27 September 2024 in Wageningen, the Netherlands.
